# Effects of high-intensity interval training on physical and cognitive function in middle-aged male mice

**DOI:** 10.3389/fragi.2025.1589730

**Published:** 2025-08-14

**Authors:** Justin C. Stephenson, Tuan D. Tran, Ted G. Graber

**Affiliations:** ^1^ Department of Kinesiology, East Carolina University (ECU), Greenville, NC, United States; ^2^ Department of Psychology, East Carolina University (ECU), Greenville, NC, United States; ^3^ Department of Physical Therapy, East Carolina University (ECU), Greenville, NC, United States; ^4^ Department of Physiology, East Carolina University (ECU), Greenville, NC, United States; ^5^ East Carolina Obesity and Diabetes Institute, East Carolina University (ECU), Greenville, IL, United States

**Keywords:** cognitive function, exercise, high-intensity interval training, physical function, treadmill training, body composition, muscle contraction, aging

## Abstract

**Introduction:**

Declining functional capacity, both physical and cognitive, is a consequence of aging. However, exercise is a promising intervention to mitigate normal age- related decline. Although numerous studies have elucidated the benefits ofexercise per se, the effect of high-intensity interval training (HIIT) on a middle-aged population is less well-studied.

**Objective:**

Our primary purpose was to assess the effect of 3 months of HIIT on physical and cognitive performance in middle-aged (17-month-old at the end) male C57BL/6J mice compared to sedentary controls (SED). We hypothesized that exercised mice would be resistant to age-related decline in cognitive and physical ability.

**Methods:**

To measure physical function, we used the well-validated comprehensive functional assessment battery (CFAB) scoring system, comprised of determinants including voluntary wheel running, inverted cling, grip test, treadmill maximum speed, and rotarod performance. We measured cognition using open field test, novel object recognition, Y-maze, and puzzle box. Additional assessments included body composition (via MRI) and in vivo contractile physiology (plantar flexor torque).

**Results:**

Training resulted in significant improvements in aerobic capacity for the HIIT group, increasing treadmill time by 28%, while the SED group demonstrated a 41.4% decline in treadmill time. However, we found no significant differences in overall cognitive function. Contrary to our previous research in other age groups, the current study found a negligible effect of HIIT on body composition.

**Discussion:**

We note that at 17 months of age, mice did not exhibit any evidence of cognitive deterioration in either group over the training period, thus potentially explaining the lack of an exercise effect. We found that HIIT had less influence on both physical and cognitive function than expected, which may be because function in this age group remains relatively stable. Future work will investigate the adult cognitive response to HIIT in older adults, at ages where there is well- documented cognitive decline.

## Introduction

In the United States alone, the percentage of adults aged 65 years or older increased by 38.6% between 2010 and 2020 (Bureau, 2020). Compared to that in 2015, the global percentage of adults aged 60 years or older is projected to reach 16.5% or more by 2030, marking an increase of more than 4% (UN, 2017). Such developments and age distribution shifts toward an older population will increase the prevalence of age-related functional (physical and cognitive) declines, along with an increased need for medical interventions, skilled nursing facilities, and in-home care.

As we grow older, mild changes in cognition are expected and considered a normal feature of the aging process (Hugo and Ganguli, 2014), also known as *normal cognitive decline*. Evidence suggests there are a variety of genetic, environmental, health, and lifestyle factors that play a role in the brain’s aging and cognitive capabilities as we age (Cadwallader et al., 2022). Certain health and lifestyle factors potentiate various forms of cognitive impairments and are modifiable risk factors (i.e., sedentariness, hypertension, obesity, etc.) that can be mitigated via exercise (Hugo and Ganguli, 2014). A 2019 meta-analysis observed significant associations between exercise-induced improvements in physical and cognitive function (Falck et al., 2019).

Regular exercise engagement throughout the lifespan may prove protective against age-related cognitive decline (ARCD), with studies showing that higher rates of exercise from early to mid-adulthood likely reduce the risk of cognitive decline later in life (Middleton et al., 2010; Yaffe et al., 2009). However, research also suggests that starting regular exercise later in life is still beneficial (Colcombe and Kramer, 2003). Studies in older adult populations have found that participating in exercise programs and increasing cardiorespiratory fitness are correlated with reductions in age-related neural changes (Erickson et al., 2011; 2010) and improvements in cognitive performance (Colcombe and Kramer, 2003; Falck et al., 2019; Freudenberger et al., 2016; Suzuki et al., 2012). Reported benefits of cardiorespiratory (aerobic/endurance) exercise on the brain include improvements in executive function and divided attention (Baker et al., 2010) and specific executive function skills such as inhibitory control and working memory (Cadwallader et al., 2022).

In this study, we examined the effects of high-intensity interval training (HIIT) on physical and cognitive performance in middle-aged male mice (aged 17 months at study completion). Previously, we demonstrated that HIIT can preserve physical function in adult (10-month-old) and older adult (26-month-old) mice (Pajski et al., 2024). HIIT is a type of exercise performed in pre-determined intervals of higher intensity interspersed with low-intensity (active rest) intervals. We hypothesized that there would be less cognitive and functional decline—as measured by the comprehensive functional assessment battery (CFAB) and a cognitive assessment battery (CAB) testing protocols—in the HIIT-exercised group (HIIT) than in the sedentary control group (SED). We measured exercise capacity and physical function with the previously-validated CFAB (Graber et al., 2021; Pajski et al., 2024; Pajski et al., 2025) and assessed cognitive function with a battery of commonly used cognitive/behavioral tests for mice (open-field test, Y-maze, novel object recognition, and puzzle box) (Ben Abdallah et al., 2011; Ohtomo et al., 2021; Sabaghi et al., 2019; Shepard et al., 2017; Szatmari et al., 2021; Wu et al., 2020). In addition, we determined the impact of HIIT on body composition (EchoMRI), muscle wet weight, and maximal isometric plantar flexor torque. We observed significant changes between the two groups’ aerobic capacity and treadmill speed but did not observe significant improvements in cognitive performance.

## Methods

### Subjects

We obtained C57BL/6J male mice (n = 16) from The Jackson Laboratory at 10 m (months) of age. Within a week of arrival and before pre-testing processes began, one subject died of natural causes, leaving 15 mice for the remainder of the study. Mice started exercise training at 14 m (middle-aged mouse) Hagan, 2017) and completed training at the middle-aged timepoint of 17 m, which is equivalent to a human in their mid-50s (see supplemental section of Pajski et al., 2024). Mice were treated humanely in accordance with guidelines approved by the ECU Institutional Animal Care and Use Committee (IACUC). Mice were group-housed under 12-h light/dark cycles at 22 °C, with *ad libitum* access to food and water. Due to aggressive behavior/fighting and resulting injuries, some mice were unavoidably singly housed during the study, as recommended by our staff veterinarians and required by the IACUC.

### Study design

**FIGURE 1:**
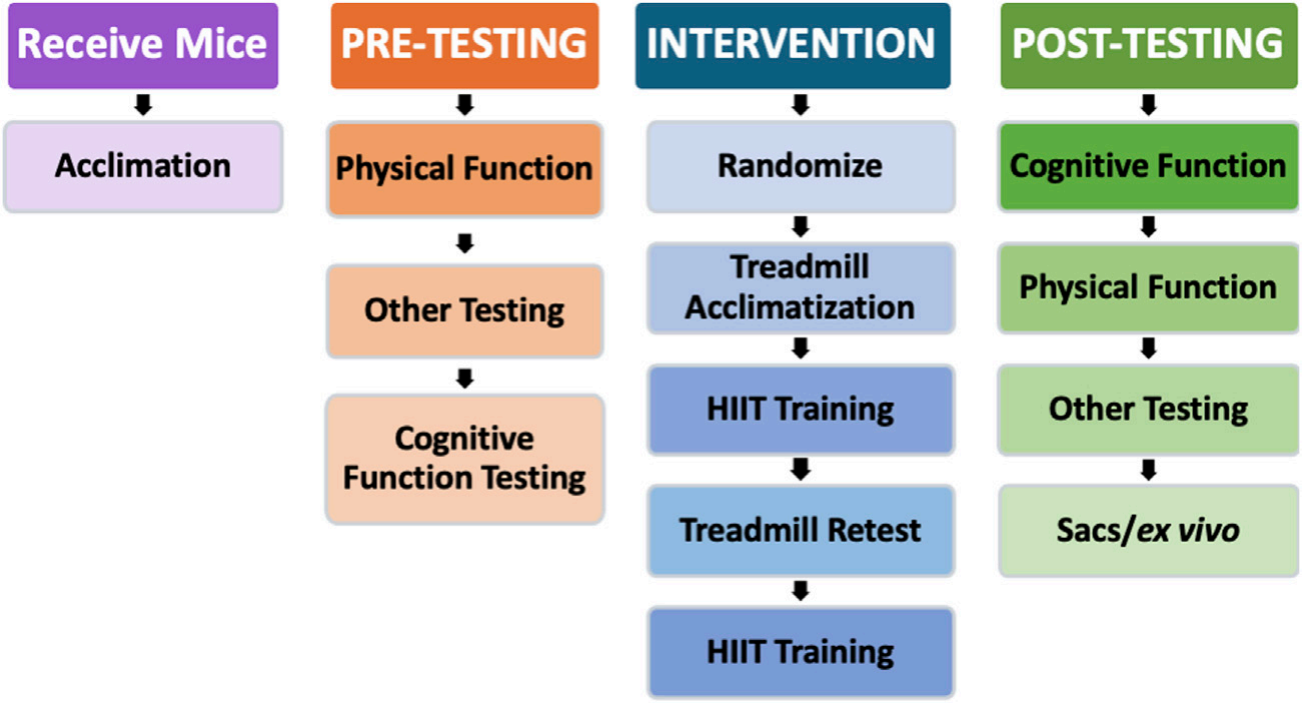
Study design. After the initial acclimation period and baseline testing, the mice began the intervention stage of the experiment. The HIIT group acclimated to treadmill training for 1 week, trained for 6 weeks, retested for maximum speed on the treadmill, and then trained for another 6 weeks. Following the HIIT intervention, all mice were retested for physical and cognitive function. Physical and cognitive function testing were reversed between pre- and post-testing to account for any biases potentially created by testing in the same order.

Figure 1 shows the study design. After an acclimation period, we completed pre-intervention performance assessments (physical and cognitive) and then randomized the mice into two groups: SED (n = 7) and HIIT (n = 8). Next, we conducted a 12-week HIIT intervention where the HIIT intervals were based on a treadmill maximum speed test. During this time, we subjected the SED mice to sham treatment. As the HIIT intervention concluded, post-testing and maintenance training commenced. Maintenance exercise training was performed during the post-testing phase to preserve any exercise-induced adaptations in the HIIT mice until testing was complete. Finally, following post-testing, we performed tissue collection for later analysis.

### Intervention

After baseline testing and data analysis, we randomized the mice into their respective groups and began intervention. The intervention period consisted of one treadmill acclimation week, followed by 12 weeks of HIIT on the treadmill. The mice completed 6 weeks of training before they were re-evaluated on a treadmill, after which, the last 6 weeks of training commenced with an adjusted baseline for their intervals based on the retest. HIIT training was three times per week, one session every other day (i.e., Monday, Wednesday, and Friday), to allow for rest and recovery between exercise sessions. During the intervention, mice were group-housed in cages with environmental enrichments, such as a block or tunnel for stimulation and play, but no means of structured exercise (i.e., running wheels) was provided, aside from general physical activity (PA). Some mice needed individually housing due to over-aggression and fighting; these housing changes were made in consultation with the veterinarians from our Department of Comparative Medicine, who oversee the animal facility.

#### High-intensity interval training

##### Acclimation and training

Before the first week of HIIT, we acclimated the mice to the treadmill and interval speed changes for 1 week (three sessions). They ran one interval on the first day, two intervals on the second day, and three intervals on the third, with warm-ups and cool-downs on each day. By the first week of training, the mice were running for three HIIT intervals at 75% of their cohort’s average maximum speed, transitioning to higher percentages and more intervals throughout the intervention. We added additional intervals as tolerated, up to a maximum of five, as the intensity/speed was also increased to the tolerance of the mice. If a mouse was unable to complete the scheduled training at the expected intensity, it moved to the next slowest group until improvements were observed. After the sixth week (midpoint) of the intervention, the mice were retested for a new maximum speed. Then, we adjusted relative interval speeds accordingly.

##### Exercise sessions

We used the average maximum speed (Speed_max_) of the mice—as measured by the baseline treadmill test—to determine percent maximum speed (%Speed_max_) for the HIIT intervals. Based on their fastest recorded speeds, we grouped the mice into exercise cohorts with similar maximum speeds. We exercised the mice 3 days/week, with one rest day between training sessions (i.e., Monday, Wednesday, and Friday) and two rest days on weekends. Each HIIT session began with a 2-min warm-up at the base speed (4 m/min), followed by 1-min intervals at sprint speed (with 30-s ramp-ups and 30-s ramp-downs, for a total of 2 min per interval), and interspersed with 1-min relative rest (walking speed). After the final HIIT interval, each session concluded with a 2-min cool-down at the base speed.

##### Sham treatment

To equalize the experiential environment of HIIT and SED mice, the SED group received a sham treatment. For this sham treatment, SED mice were placed on the treadmill each day that HIIT training was conducted. SED mice did not exercise, but they explored the unmoving treadmill for the same total time as each HIIT session, with the shock grid activated.

### Performance assessments

The same investigators performed the physical function and cognitive assessments throughout the study, with baseline and end-point assessments in the same order and at the same time of day. We used maintenance exercise training to maintain adaptations throughout the post-testing period.

### Physical function assessments

#### Functional performance

We assessed mouse physical function and exercise capacity via CFAB pre- and post-intervention. We previously validated the CFAB for male mice at three different ages (adult 6 m and older adult at 24 and 28 m) and longitudinally in male and female mice across the lifespan (Graber et al., 2021; Pajski et al., 2024). In summary, we tested the mice using a series of commonly used well-validated determinants, including grip meter (forelimb strength), inverted cling (full-body strength/endurance), voluntary wheel running (VWR; volitional exercise and individual activity levels), rotarod (overall motor function, gait speed, balance/coordination, and power generation), and treadmill (maximum running speed and endurance/aerobic exercise capacity). The methodology for the determinants comprising CFAB has been previously described (Graber et al., 2021; Pajski et al., 2024).

### CFAB data analysis

Traditionally, CFAB data analysis uses a reference group of 6-month-old mice (mean and standard deviation; SD), and test results for each mouse are standardized (difference in SD from previously published 6-month means) and summed together to quantify a composite CFAB score, a single numeric value representative of overall physical function capacity (Graber et al., 2023; 2021; Pajski et al., 2024). In the current study, baseline standardization was based on the pre-intervention mean and SD of the entire sample (*n* = 15), assessed prior to randomization. Individual mouse scores were then compared to this baseline. We compared the differences between pre- and post-testing (ΔCFAB = CFAB_post_ – CFAB_pre_), similar to our frailty intervention assessment value (FIAV), as previously explained (Graber et al., 2015).

### Other physical measures

#### Body and muscle mass

We determined body composition (fat percentage, fat%) from pre- to post-intervention using an EchoMRI-700d. The EchoMRI-700™ (Echo Medical Systems) is a quantitative nuclear magnetic resonance system that provides precise whole-body composition measurements *in vivo*.

#### *In vivo* contractile physiology

We determined plantar flexor torque using the Aurora Whole Mouse 3-in-1 Physiology Suite (Aurora Scientific), as previously described (Brightwell et al., 2021; Graber et al., 2021). In brief, each mouse was anesthetized on a 37 °C heated platform to maintain body temperature using ∼3% isoflurane delivered at 1.5 L/min of O_2_ via a VetEquip Vaporizer and nosecone, effectively eliminating conscious control of skeletal muscles. We positioned the knee at 90°, with the tibia aligned parallel to the platform. The femur was stabilized by clamping the lateral and medial epicondyles to prevent leg movement while still allowing for free movement below the knee. We set the foot into a footplate connected to a force transducer and then adjusted the height to firmly set the heel into the bottom of the plate. Using subcutaneously placed needle electrodes, we determined the optimal location and current needed to produce a maximum torque twitch. This current and needle placement were maintained during a torque/frequency curve (a single pulse and then 10, 40, 80, 100, 120, 150, 180, and 200 Hz) to determine the maximum tetanic isometric torque of the plantar flexor muscles (triceps surae).

### Cognitive function assessments

#### Cognitive performance

We assessed cognitive function with our cognitive assessment battery (CAB) pre- and post-intervention. We determined cognitive performance through the application of memory, behavioral, and executive function tasks. The tests included in CAB were as follows: open-field test for anxiety, locomotor, and exploratory behavior (Creighton et al., 2019; Szatmari et al., 2021); Y-maze for exploratory behavior and spatial working memory (Sabaghi et al., 2019); novel object recognition (NOR) for exploratory behavior and long-term memory (Creighton et al., 2019; Szatmari et al., 2021); and puzzle box for memory and executive function (Ben Abdallah et al., 2011; Shepard et al., 2017). We recorded all cognitive/behavioral assessments using a GoPro Hero 6 Black for later analysis and data quantification. We analyzed CAB outcome measures individually. The CAB determinants are briefly explained as follows.

#### Open field

The Open field (OF) test is a commonly used behavioral test for assessing general locomotor activity, anxiety-like behavior, and exploratory tendencies in mice (Creighton et al., 2019; Szatmari et al., 2021). The testing arena was a 58 × 58 × 40 cm box, made of a non-abrasive plastic, with an open top for direct lighting and video recording. Before testing, we assessed light distribution using the Light Meter LM-3000 to ensure uniformity of brightness (750 ± 10 lux) across the testing arena. We applied direct lighting using a single LED lamp positioned over the center of the arena. The outcome measures for OF were the number of entries into the center and the time (s) spent in the perimeter.

#### Y-maze

The Y-maze assessed spatial working memory, as described in the literature (Sabaghi et al., 2019). We used a custom-built symmetrical Y-shaped maze with a non-reflective, neutral-colored surface (beige); the details are provided in Supplementary Information. In brief, we positioned each mouse in one arm of the maze (designated Arm A), facing the center, and allotted them 8 min of uninterrupted exploration time. We defined entry into an arm as the animal having all four paws inside the arm. We assessed locomotor activity via total arm entries, and any latency to leave the starting arm was an indication of emotionality-related behavior. The percentage of spontaneous alternation performance (%SAP) was our outcome measure for this test. We defined a spontaneous alternation (SA) in this experiment as sequential entries into all three arms in overlapping triplet sets (i.e., ABC, BCA, CAB, or vice versa), as shown in Supplementary Figure S3. We calculated the %SAP as the ratio of total alternations to possible alternations (%SAP = SA/[total arm entries – 2] x 100).

#### Novel object recognition

We administered NOR to assess long-term memory and exploratory behavior in mice (Creighton et al., 2019; Szatmari et al., 2021). Discrimination of novel versus familiar stimuli requires intact perceptual systems. Therefore, if a mouse spends more time exploring a novel object (NO) compared to a familiar object (FO), it is indicative of an intact memory (Creighton et al., 2019). We calculated a discrimination ratio (DR) to quantify novelty preference. We did so by subtracting the time (s) spent exploring the FO from the time spent exploring the NO and then dividing the difference by the total object exploration time (s; DR = (NO – FO)/total exploration). DR was the outcome measure for the NOR test.

#### Puzzle box

The puzzle box is a commonly used test that is designed to assess executive function skills in mice via working memory and problem-solving requirements. We based our adaptation on previously used versions (Ben Abdallah et al., 2011; Shepard et al., 2017). For this adaptation of the puzzle box assessment, a PVC pipe connects the big OF arena to a much smaller “puzzle box” (17.3 × 21 × 17.6 cm). The OF arena was the open, brightly illuminated starting area, while the smaller, darker puzzle box was the objective area. To access the objective area, subjects must climb into the tunnel and make their way across.

We began the puzzle box tasks by positioning each mouse in the center of the wall, directly across from the puzzle box access. We released the mice and started a timer. The outcome measure was the latency to complete each objective. We used a treat/prize (i.e., an unsalted walnut, almond, or plain Cheerio) as an additional incentive. With each test, we modified the access point to increase the difficulty of reaching the puzzle box. First, we evaluated the mice without any obstacle to accessing the puzzle box (day 1). Next, we added an obstruction, blocking the exit of the PVC pipe, which the mice had to simply knock down to gain access to the puzzle box (day 2). The last task had two trials (T1 and T2), and for each trial, the entrance point faced a different direction (day 3). For analysis of this test, we used a 0–1 scoring system based on the completion of certain objectives (i.e., entering the tunnel and removing the barricade). Subjects were allotted a 0 for each objective that they failed to complete or a 1 for each objective successfully completed. The outcome measure for this test was the total score achieved on all objectives.

### Statistical analysis

We used Student’s independent samples, paired t-tests, and 2 × 2 ANOVA, as appropriate, to compare dependent variables with the results reported in the appropriate tables, alongside the mean, SD, SEM, effect size (Cohen’s D or η^2^), skew, kurtosis, and the results of the Kolmogorov–Smirnov and Shapiro–Wilk tests for normality (details are reported in online-only Supplementary Datasheets S1–6 and Table 1). We used independent-samples t-tests to compare the mean differences in CFAB and CAB performance scores between the HIIT and SED groups. We compared changes within the groups using paired sample t-tests. We also assessed CFAB functional determinants using a 2 × 2 mixed-design ANOVA (see results for more details). Differences were deemed significant at *p* < 0.05. Data were expressed as the mean ± SE (standard error), unless otherwise indicated, with the effect sizes reported as appropriate.

**TABLE 1:** Main findings: statistics: *p*-value (*p*-val) for between groups from independent t-test and for within groups from paired t-test. *p*-val is given in bold if significant. KEY: HIIT, high-intensity interval training group; SED, sedentary control group; mN*m/gbm, milliNewtons-meters normalized to grams of body mass; pre, prior to the intervention period; post, following intervention period.

Between groups						
Test	Variable	Units	Group	Mean ± SE	Effect size	*p*-val
Treadmill	Time to failure	Seconds	HIIT	630.75 ± 43.06	2.883	**<0.001**
			SED	308.14 ± 37.48		
		%change	HIIT	36.42 ± 14.80	1.974	**0.002**
			SED	−34.68 ± 10.46		

Within groups							
Test	Variable	Units	Group		Mean ± SE	Effect size	*p*-val
Treadmill	Treadmill time/aerobic capacity	Seconds	SED	Pre	525.86 ± 70.23	1.475	**0.008**
				Post	308.14 ± 37.48		
			HIIT	Pre	492.63 ± 49.72	−0.923	**0.035**
				Post	630.75 ± 43.06		
Body composition	Body mass	Grams	SED	Pre	35.46 ± 1.72	−1.385	**0.019**
				Post	40.08 ± 3.33		
			HIIT	Pre	35.51 ± 1.62	−1.118	**0.016**
				Post	39.17 ± 2.59		
	Fat%	%	SED	Pre	19.72 ± 4.06	−1.311	**0.013**
				Post	26.11 ± 3.17		
			HIIT	Pre	22.00 ± 3.61	−2.169	**<0.001**
				Post	27.28 ± 3.48		
Plantar flexor torque	Normalized torque	mN*M per gbm	HIIT	Pre	0.86 ± 0.064	0.076	0.873
				Post	0.86 ± 0.067		
			SED	Pre	0.83 ± 0.067	0.612	0.243
				Post	0.74 ± 0.056		
Open field	Time spent in perimeter	Seconds	SED	Pre	435.29 ± 5.30	−0.863	0.063
				Post	450.71 ± 7.37		
			HIIT	Pre	433.38 ± 5.29	−1.119	**0.016**
				Post	458.63 ± 5.08		
Y-maze	Spontaneous alternations (SAs)	Count	SED	Pre	14.43 ± 1.02	−1.330	**0.013**
				Post	19.14 ± 1.16		
			HIIT	Pre	11.88 ± 1.37	−1.63	**0.002**
				Post	18.00 ± 1.10		
	Spontaneous alternation %	SA/(total-2)	SED	Pre	63.11 ± 3.22	−0.110	0.781
				Post	64.05 ± 4.24		
			HIIT	Pre	60.15 ± 3.34	0.330	0.381
				Post	56.75 ± 2.08		
	Total arm entries	Count	SED	Pre	25.00 ± 1.45	−1.501	**0.007**
				Post	32.29 ± 2.00		
			HIIT	Pre	22.00 ± 2.53	−1.881	**0.001**
				Post	33.88 ± 1.89		

## Results

Further details can be found in online-only Supplementary Datasheets S1–6 and Table 1.

### Physical function (CFAB): improved aerobic capacity from HIIT

We determined physical function and exercise capacity pre- and post-intervention using CFAB. Note that all CFAB determinants were normally distributed, as we have previously determined and published (Graber et al., 2021), with the exception of inverted cling, which we transformed to log10 to meet the criteria of normality for our statistical tests.

**FIGURE 2:**
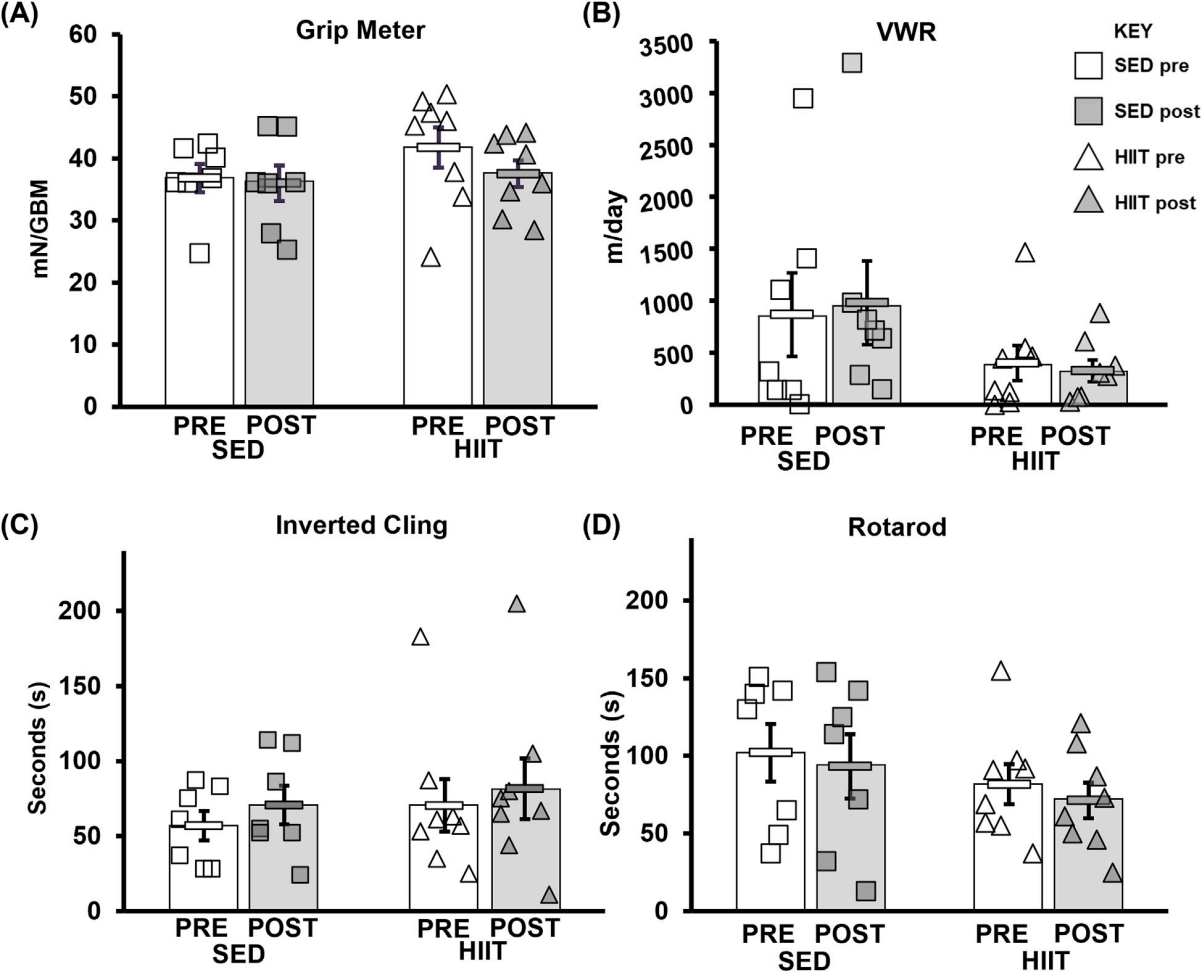
CFAB determinants. **(A)** Grip meter. **(B)** VWR. **(C)** Inverted cling. **(D)** Rotarod. From pre- to post-intervention, none of these tests demonstrated significant training effects. KEY: mN/GBM, milliNewton per gram of body mass. SED, sedentary control group; HIIT, high-intensity interval training group; PRE, baseline before the intervention period; POST, value after the intervention period.

There were no significant changes (2 × 2 mixed ANOVA [two groups: SED and HIIT; and two time points: pre- and post-intervention]) in grip meter (strength), inverted cling (overall strength/endurance), voluntary wheel running (volitional exercise), or rotarod (overall motor function) with training either between or within subjects compared to sedentary mice. More details can be found in Supplementary Datasheet S1 and Figure 2.

**FIGURE 3:**
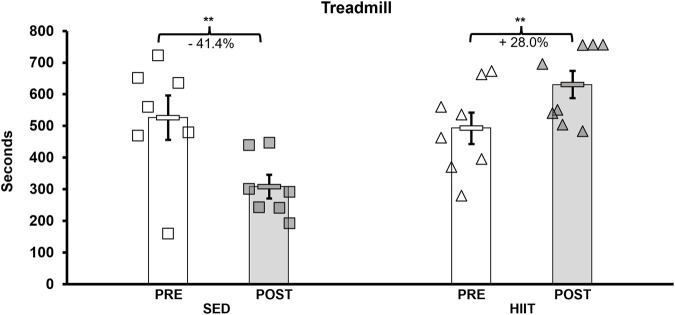
Treadmill maximum speed test. Pre-intervention testing revealed no significant difference in aerobic capacity, and after training, HIIT aerobic capacity increased (+138.1 s) and SED decreased (−217.7 s). KEY: SED, sedentary control group; HIIT, high-intensity interval training group; PRE, baseline before the intervention period; POST, value after the intervention period; ** = *p* < 0.01.

**FIGURE 4:**
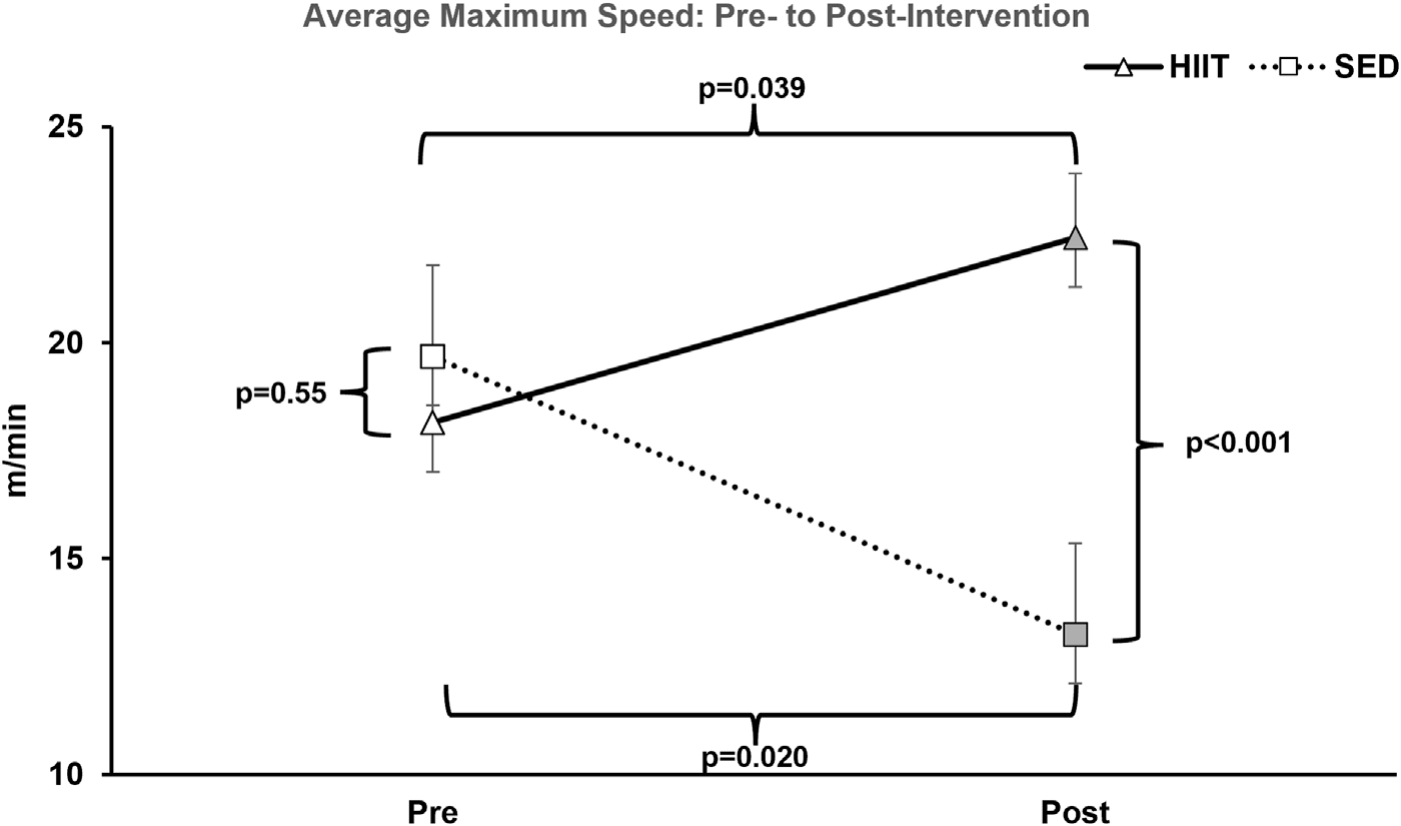
Average maximum treadmill speed. At pre-intervention testing, there was no significance observed between groups. However, after the training period within-group, HIIT significantly increased and SED decreased; between-group HIIT > SED. KEY: SED, sedentary control group; HIIT, high-intensity interval training group; PRE, baseline before the intervention period, POST, value after the intervention period.

To assess changes in aerobic capacity and running speed, we administered the treadmill maximum speed test pre- and post-intervention for both groups. In the HIIT group, treadmill time significantly increased by 28.0% from pre- to post-training, while the SED group showed a decline in performance by 41.4% (2 × 2 ANOVA within-subjects interaction effect of time*groups: F = 21.381, *p* < 0.001, and partial η^2^ = 0.622; between-subjects effect of time*groups: F = 5.572, *p* = 0.035, and partial η^2^ = 0.300). On average, the HIIT group increased treadmill time by 138.1 s, while the SED group declined by 217.7 s (see Supplementary Datasheet S1 and Figure 3) from pre- to post-intervention (between-group *post hoc* testing using an independent-samples t-test t = 5.572 and *p* < 0.001; within-subjects *post hoc* paired-samples t-tests showed that both groups experienced significant changes with large effect sizes: SED t = 3.901, *p* < 0.008, and Cohen’s d = −1.475; HIIT t = 2.612, *p* = 0.035, and Cohen’s d = 0.923). In addition, between the groups, the mean maximum speed was equal during pre-testing (*p* = 0.555) but was significantly altered from pre- to post-training (*p* = 0.00008) (see Figure 4). Within the groups, the mean maximum speed increased in the HIIT group (*p* = 0.038) and decreased in the SED group (*p* = 0.019). Overall physical function, as measured by the CFAB, did not alter from pre- to post-training (within-subjects effect of time*groups: F = 0.070, p = 0.795, and partial η^2^ = 0.005; between-subjects effect of time*groups: F = 0.037, p = 0.851, and partial η^2^ = 0.003). Further details can be found in Supplementary Figure S1 and Supplementary Datasheet S1.

### Other measurements

#### Body composition

**FIGURE 5:**
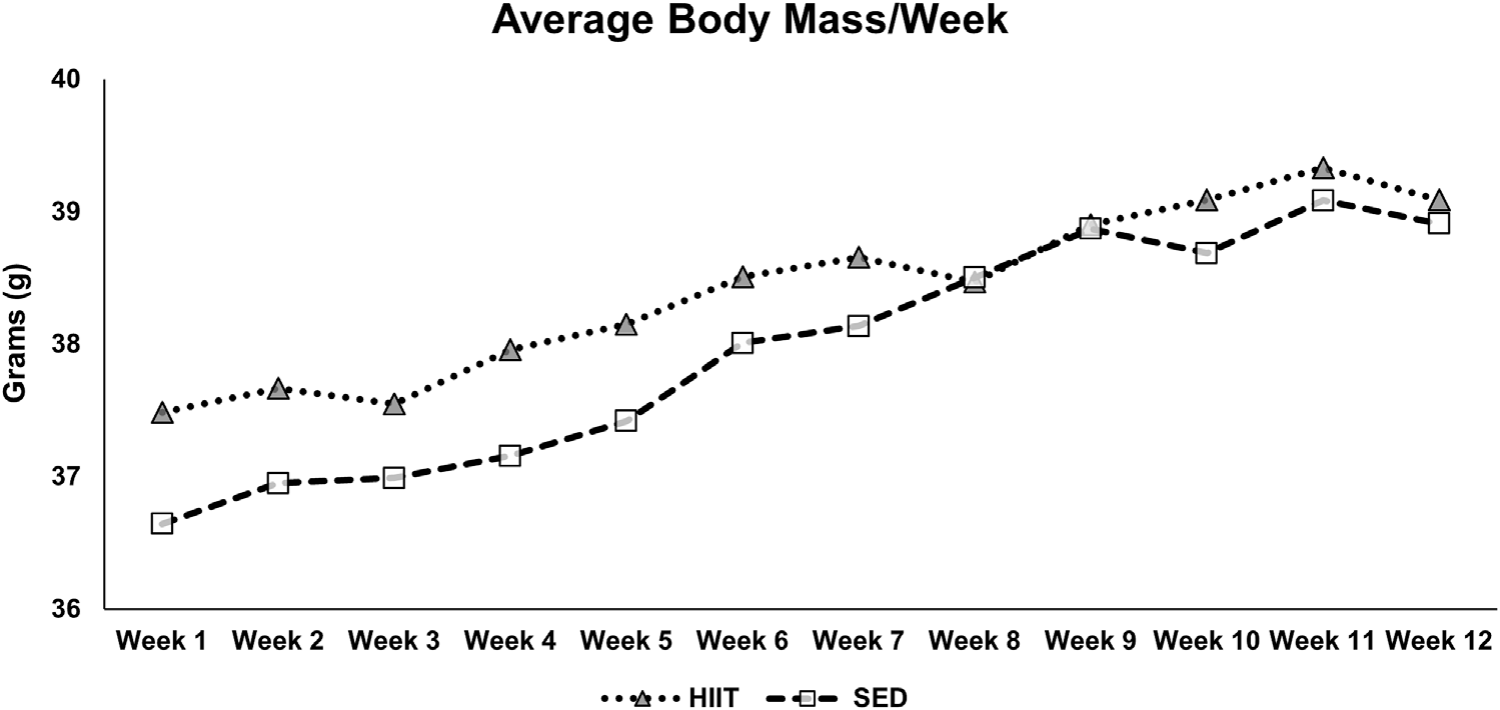
Average body mass by week. The mice were exercised or exposed to sham treatment for 3 days/week. Every week, before their third session, the mice were weighed, and the average body mass was calculated. KEY: SED, sedentary control group; HIIT, high-intensity interval training group.

We measured the body mass (grams; g) weekly throughout the intervention (see Figure 5), prior to each EchoMRI, and at euthanasia. There was no difference in any of the measurements prior to training. The within-group differences in body mass, fat mass, and fat% measured by EchoMRI were significantly greater from pre- to post-training (2 × 2 repeated measures ANOVA: F = 20.062, 46.845, and 35.899, respectively; all *p* < 0.001). Lean mass (within groups) tended to decrease in SED and yet remained the same in HIIT from pre- to post-training (2 × 2 repeated measures ANOVA: F = 3.551 and *p* = 0.082). Between groups, the lean mass difference had a strong effect size (−0.746) though it was not statistically significant (independent samples t-test, *p* = 0.173), potentially due to large individual variability. Further details can be found in Supplementary Datasheet S2 and Figure 5.

#### Muscle mass

At euthanasia, we collected the gastrocnemius (GAS), plantaris (Plant), tibialis anterior (TA), extensor digitorum longus (EDL), soleus (SOL), and heart. We blotted the tissues dry and weighed them (grams; g) before flash-freezing for later analysis. Between-groups analysis (independent-samples t-test) showed no significant differences for each muscle or the total muscle mass (composite of GAS, Plant, TA, EDL, and SOL). Further details can be found in Supplementary Datasheet S3.

#### *In vivo* contractile physiology

We determined the maximum isometric plantar flexor torque before and after the intervention period. There were no significant differences between the groups at pre-intervention testing. We found no significant changes within groups (paired samples t-test) in either the maximum torque (mN*m) or normalized torque (mN*m/grams of body mass; paired samples t-test: SED t = 1.369, p = 0.243, and Cohen’s d = 0.612; HIIT t = 0.170, p = 0.873, and Cohen’s d = 0.076) following training. Further details can be found in Supplementary Figure S4.

### Cognitive function

We measured different parameters of cognitive function using four different tests administered pre- and post-intervention and then analyzed the results using independent-samples t-tests between groups and paired samples t-tests within groups. Further details can be found in Supplementary Datasheet S5, Table 1.

#### Open field

For the OF test, we counted entries into the center and the time spent in the perimeter. Within the HIIT group, pre- to post-intervention changes demonstrated a significant increase in time spent in the perimeter (*p* = 0.016, effect size 1.2), which is indicative of greater anxiety-like behavior. The SED group tended (*p* = 0.063, effect size 0.86) toward a similar result. However, there were few defecation and urination events during both pre- (four total for all mice) and post-testing (none), indicating limited evidence of anxiety in this regard. The details can be found in Supplementary Figure S2.

#### Y-maze

The Y-maze assesses subjects’ spatial reference memory by measuring the number of spontaneous alternations (SAs) made in the allotted test time and converting the SAs into a percentage of the total number of arm alternations (%SAP). The number of SAs increased significantly in both groups (within groups: HIIT, *p* = 0.002, effect size 1.63; SED, *p* = 0.013, effect size 1.33 from pre- to post-intervention). However, the total arm entries also increased significantly in both groups (within groups: HIIT, p = 0.001, effect size 1.99; SED, p = 0.007, effect size 1.50), thus resulting in no significant improvements in %SAP. Although the total arm entries increased in both groups, the increase was greater in the HIIT group (54% versus 31.2% in SED). The details can be found in Supplementary Figure S3.

#### Novel object recognition

There were no significant changes in NOR between groups (p = 0.757) from pre- to post-training. No changes were observed within the groups (SED p = 0.20; HIIT p = 0.502). The details can be found in Supplementary Figure S4.

#### Puzzle box

We did not find significant overall changes in the puzzle box tests between groups after training with our total point system (*p* = 0.689, effect size 0.212), and there was no difference in pre-testing (*p* = 0.452, effect size 0.40). The total point system also showed no changes within-group from pre- to post-intervention (SED *p* = 0.356, effect size 0.38; HIIT *p* = 0.32, effect size 0.13). However, for the blocked exit task, the between-group analysis showed that the SED group was significantly faster at *entering the tunnel* (*p* = 0.036, effect size 1.33) pre-intervention, but this was not replicated in post-testing (p = 0.306, effect size 0.55). Although between-group analysis showed significance for other test variables collected, none fit normality. For example, in the differing entrance task, analyses showed that the SED group was significantly better at locating the second (T2) entrance during the pre-intervention assessment (*p* = 0.005), but this advantage was not observed post-intervention (*p* = 0.904). The details can be found in Supplementary Figure S5.

### Exercise intensity/work

#### HIIT intensity and work increased through middle age

Throughout the intervention, the HIIT group’s exercise intensity and work performed increased relative to how each mouse was responding to their current exercise load. Over the course of the HIIT intervention, exercise intensity (%Speed_max_) increased by an average of 14.36%. Between the midway point (week 6) treadmill retest and the last day of training (week 12), exercise intensity increased by an average of 15.27%. We calculated the total amount of work (m*gbm, meters*grams of body mass) for each HIIT session performed. The average significant difference (*p* < 0.05) in power produced (work performed per minute) between each subject’s first and last HIIT session was equal to 40.32 (m*g)/min. This indicates evidence of exercise adaptation.

## Discussion

We designed the current study to determine the pre- to post-training effects of a 12-week HIIT treadmill protocol (Pajski et al., 2024) on the functional and cognitive performance in middle-aged C57BL/6J male mice compared to that in a sedentary control group. Overall, CFAB did not change with exercise in this population, though we observed significant improvements in the HIIT aerobic capacity and treadmill speeds, with a corresponding decline in SED. We observed significant increases from pre- to post-training in both HIIT and SED for body mass, fat mass, and fat % (within groups) and a strong effect size for lean mass difference between groups (HIIT retains lean mass, though not significantly). Finally, we did not detect any significant cognitive changes between the two groups. However, in the Y-maze test, there was an overall increase in exploratory behavior (more SA and more arm entries), yet there was no change in the ratio, indicating no increase in memory function. The increased level of exploratory behavior may be indicative of reduced anxiety during the repeated measurement, perhaps due to memory of the prior test or increased handling leading up to the second assessment.

### HIIT

In recent years, HIIT has been popularized as a safe and time-conscious alternative mode of exercise. HIIT exercise research has reported positive effects on several chronic diseases (Askim et al., 2014; Molmen-Hansen et al., 2012; Rose et al., 2020; Støa et al., 2017) often associated with cognitive decline/diseases in older adults (Annual Congress, 2020). Furthermore, even short-term (6 weeks) HIIT has produced physiological and physical fitness improvements similar to, and better than, endurance and/or resistance training in middle-aged men (Callahan et al., 2021).

We adapted our 12-week treadmill HIIT protocol from previous studies (Pajski et al., 2024; Seldeen et al., 2018). While Seldeen et al. (2018) used mice aged 22 m, the findings from Pajski et al. (2024) were based on adult mice aged 6 m–10 m and older mice aged 22 m–26 m. Thus, there remains a gap in the literature for the 14 m–17 m (middle age) age range.

Graber et al. (2021) validated the use of CFAB in male mice at 6 m, 24 m, and 28 + m of age, observing an overall age-related physical function decline. In the current study, we detected no significant changes in CFAB or the CFAB determinants of grip meter, inverted cling, VWR, and rotarod. This contrasts with prior findings of significant improvements in, or preservation of, physical function (Pajski et al., 2024; Seldeen et al., 2019; Seldeen et al., 2018). However, Seldeen et al. (2019); Seldeen et al. (2018) did not observe improvements in rotarod for the HIIT group in their 2018 study, nor did they observe significant changes in rotarod or inverted cling for either group (SED or HIIT) in their 2019 study. Notably, these studies investigated different age groups than the current study. In a recent work, a relative plateau in functional capacity between 12 m and 18 m in male C57BL/6 found by Pajski et al. (2024) indicated a stability of function, instead of decline, in early-to-later middle-age, which might partly explain our results. Although overall CFAB scores did not improve with training, there was a marked improvement in aerobic capacity (treadmill time) and treadmill speeds in the HIIT group, while the sedentary control mice exhibited a decline. This finding is consistent with prior studies (Callahan et al., 2021; Pajski et al., 2024; Seldeen et al., 2019; Seldeen et al., 2018) in both animals and humans.

Contrary to our hypothesis, neither group showed a decline in cognitive function, nor did exercise result in any improvement. With no decline in either group, there was no preservation of function either. In the literature, there appears to be a correlation between age and cognitive performance in C57BL/6 male mice (Daneshjoo et al., 2022; Pettan-Brewer et al., 2013). Pettan-Brewer et al. did not demonstrate changes measured by the radial water tread maze in male mice (measured at 4, 12, 20, and 28 months of age) between 12 m and 20 m. Daneshjoo et al. (2022) showed a naturally occurring age-related cognitive decline using the same measurement in male mice, with statistically significant differences observed between the younger (4 m) and older (28 m) age groups; and again, although the 12 m–20 m age group was not significantly different, there was a trend. However, age-related cognitive impairments in memory and learning behavior have been identified in C57BL/6 mice at 10 m and 14 m in age, respectively (Fouquet et al., 2011; Mechan et al., 2009). Furthermore, regarding C57BL/6 mice, a review study by Radulescu et al. (2021) revealed that notable deficits in cognitive function can be observed as early as 12 m–13 m and become progressively worse with age, with clear deficits apparent at 17 m of age (Buscher et al., 2017; Lamberty and Gower, 1990). Thus, age-related cognitive function patterns during normal aging in BL/6 mice, particularly in unexamined age-groups and through longitudinal analysis, warrant further investigation.

In human research, there are mixed reviews on whether exercise, or specific types of exercise, has any significant effect on cognition. A review by Gates et al. (2013) on the effects of exercise on cognitive function in older adults (65–95 years old) with mild cognitive impairment (MCI) revealed limited evidence supporting exercise-induced functional improvement (Gates et al., 2013). However, in 2022, results from a comparison study on the effects of HIIT and moderate-intensity continuous training (MICT) on cognitive function indicated that exercise alone could promote cognitive function independent of the exercise type (de Lima et al., 2022). A meta-analysis by Cammisuli et al. (2017) reported aerobic exercise as beneficial for patients with mild cognitive impairment (MCI), but they observed only moderate effects of aerobic training on global cognition, inhibitory control, logical memory, and divided attention (Cammisuli et al., 2017). Subsequently, Cammisuli et al. (2018) reported limited evidence for improvements in AD patients’ cognition from aerobic exercise (Cammisuli et al., 2018). However, other reviews and meta-analyses present evidence of the efficacy of exercise training to improve cognition, with resistance training often observed as a superior intervention in some cognitive domains and relative exercise intensity playing a central role, though very limited information on HIIT is available (Bliss et al., 2021; Gallardo-Gómez et al., 2022; Huang et al., 2022; Zhang et al., 2023).

The literature suggests that exercise intensity may be a critical factor in maintaining/improving memory, with higher intensities reportedly improving memory in sedentary older adults (Kovacevic et al., 2020). Contrary to our findings, where significant aerobic capacity improvements in the HIIT group showed no cognitive improvements, Kovacevic et al. (2020) found a significant correlation between increased cardiorespiratory fitness and memory improvements in humans aged above 60 years. However, by the end of the study, our mice were only equivalent in age to humans in their mid-50s.

Thus, the absence of cognitive changes—as a result of exercise intervention—observed in the current study could be attributed to multiple confounders ranging from the type of exercise, the volume/intensity/length of the protocol, the ages of the mice, or even housing conditions since social isolation in middle-aged mice can exacerbate anxiety-driven behaviors (Magalhães, et al., 2024). Additionally, there could be a ceiling and/or plateau effect in cognitive changes from 12 m to 17 m in age, where early cognitive impairment in C57BL/6 mice may begin before the start of our age range, but clear deficits are not evident until 17 m or later (Buscher et al., 2017; Daneshjoo et al., 2022; Fouquet et al., 2011; Mechan et al., 2009; Pettan-Brewer et al., 2013; Radulescu et al., 2021). The specific tests we used (open-field test, puzzle box, Y-maze, and NOR) have been successful in determining cognitive changes in various mouse models, including middle-aged mice. However, most studies in the literature showing efficacy to detect cognitive change in middle-aged mice are either cross-sectional (comparing younger adults to middle-aged adults, for example) (Magalhães, et al., 2024; Morgan, et al., 2018) or compare interventions/models with the expectation of extreme cognitive effects (e.g., NOR: Alzheimer’s model, Lourenco, et al., 2019; y-maze: ischemia/reperfusion model, Ohtomo, et al., 2021; open field/NOR: lipopolysaccharide model, Dockman, et al., 2022; and puzzle box: high-fat diet, Williams, et al., 2020). Only a limited number of short-term longitudinal studies exist in this age group in wild-type mice, although some demonstrate the ability of the tests to detect changes when they exist (Tsai, et al., 2018). Thus, our mice may have already been mildly affected by the cognitive effects of aging, with further decreases that are not evident at our study endpoint—especially during the relatively short, 3-month intervention period. This emphasizes the need for further research on exercise interventions that start in middle age and extend into older adulthood, when clear cognitive deficits typically emerge, or on the effects of lifelong exercise.

### Exercise and body composition

We observed significant increases in body mass, fat mass, and fat % within groups from pre- to post-training and a strong effect size for lean mass difference between groups, but it was not significant. Using older-adult (24 m) mice, Seldeen et al. (2018) reported significant declines in fat % for their SED mice, while their HIIT group exhibited no such decline, maintaining greater fat % than the control group. Previously, we reported marked fat% declines in both 26-m exercise groups (voluntary wheel running and HIIT) compared to increases in controls, while that in the 10-m groups all increased, although we significantly mitigated increased fat gain with exercise (Pajski et al., 2024). Natural body composition patterns observed in humans and rodents may explain the findings of Seldeen et al. (2018), Pajski et al. (2024), and the current study. A recent review found that, on average, peak fat mass in mice that are provided food *ad libitum* occurs between 12 m and 24 m, while researchers observed fat mass decline between 17 m and 24 m (Nagy and Pappas, 2019). As such, it was reasonable for our mice to continue gaining fat regardless of training or sedentary behavior.

### Caveats

One limitation of the current study is that due to within-house fighting, certain mice in both groups required individual housing, while the remaining were group-housed. According to a meta-analysis reviewing common methodological issues in animal research investigating the effects of exercise on cognition, singly housed rodents may suffer from social isolation (Hatchard et al., 2014). The study found that social isolation was associated with a greater effect of exercise on cognitive performance. The greater effect of exercise observed in socially isolated animals could be attributed to reduced environmental impoverishment as singly housed mice do not compete with cage mates for environmental nourishment (activity space, food, bedding, etc.), or it could be attributed to innate species-specific mannerisms where the male mice compete for control in each housing environment. As explained by Mechan et al. (2009), female mice may be more suitable for aging studies given that they can be group-housed for lengthy periods and placed in diverse groups, unlike male mice. Due to randomization and the fighting that occurred before and after randomization, 42.85% of the SED mice were singly housed, while just 25% of the HIIT mice were singly housed. This limitation could potentiate the lack of cognitive differences observed between the groups as it may have mitigated the overall cognitive performance of HIIT compared to that of SED. However, as previously noted, social isolation in middle-aged mice has been found to increase anxiety behaviors during open-field tests and reduce spatial memory performance in the Morris water maze (Magalhães, et al., 2024). Group-housed mice also tend to demonstrate less anxious or depressed behaviors (Liu, et al., 2013). Nevertheless, in the wild, adult male mice do not associate socially but rather exist in family units consisting of a single adult male, a number of female mice, and their offspring, and thus, aggressive behavior between group-housed adult male mice remains a constant challenge to bridge and reconcile animal welfare and study-design considerations (Kappel, et al., 2017). An optimal solution, which should be the focus of future work, would be to house individual adult male mice with age-matched ovariectomized female mice, providing companionship without innate evolutionary territorial aggression that could confound outcomes. We believe that the effects of social isolation can also be context-dependent since our prior work has not consistently demonstrated negative outcomes under similar conditions. Thus, we conclude that further research is needed regarding the interaction of social dynamics, housing, exercise, and cognition in adult, middle-aged, and older adult male mice.

Additionally, the current study only used male mice of a single strain and age-range, limiting generalization of the findings to other ages, strains, or female mice. Furthermore, our small sample size per group may have had insufficient power to detect potential subtle changes in cognition in this age group due to the sensitivity of the tests and considering high individual variability in functional testing. In our future work, we will study different age groups and female cohorts in larger numbers.

## Conclusion

We did not observe statistically significant changes between groups, pre- to post-intervention, in grip meter, inverted cling, VWR, rotarod, or overall function (ΔCFAB). However, the HIIT group showed significant increases in aerobic capacity and treadmill time, while those in the SED group exhibited significantly declines. Thus, we observed HIIT to be effective at improving aerobic capacity and running speed in middle-aged mice. We observed no significant between-group differences for any of the cognitive measures assessed. However, SED mice had no changes in cognition either; thus, we cannot conclude that HIIT is ineffective at preserving cognition in middle age. The lack of cognitive changes observed could be due to the age of the mice as they may not have started experiencing extensive age-related cognitive decline yet, which would mitigate any potential effects of the HIIT intervention to preserve function. Lack of sensitivity in the tests, the small group size, and large individual variability may also have masked subtle changes. Therefore, further research is needed to determine the interactive effects of exercise and cognition. Specifically, future research should focus on a broader range of ages in mice and assess whether biological factors such as sex and strain contribute to these differences. Our laboratory is currently addressing this research with older subjects and female mice. Additionally, with a greater sample size, future research should investigate the relationship between social isolation and exercise influence on cognition using the various CAB tests in both male and female subjects of different age groups.

## Data Availability

The original contributions presented in the study are included in the article/Supplementary Material, and further inquiries can be directed to the corresponding authors.
